# Bacteriophage Lysin Mediates the Binding of *Streptococcus mitis* to Human Platelets through Interaction with Fibrinogen

**DOI:** 10.1371/journal.ppat.1001047

**Published:** 2010-08-12

**Authors:** Ho Seong Seo, Yan Q. Xiong, Jennifer Mitchell, Ravin Seepersaud, Arnold S. Bayer, Paul M. Sullam

**Affiliations:** 1 Division of Infectious Diseases, Veterans Affairs Medical Center and the University of California, San Francisco, California, United States of America; 2 Division of Infectious Diseases, Harbor-UCLA Medical Center, Torrance, California, United States of America; 3 University College, Dublin, Ireland; Harvard Medical School, United States of America

## Abstract

The binding of bacteria to human platelets is a likely central mechanism in the pathogenesis of infective endocarditis. We have previously found that platelet binding by *Streptococcus mitis* SF100 is mediated by surface components encoded by a lysogenic bacteriophage, SM1. We now demonstrate that SM1-encoded lysin contributes to platelet binding via its direct interaction with fibrinogen. Far Western blotting of platelets revealed that fibrinogen was the major membrane-associated protein bound by lysin. Analysis of lysin binding with purified fibrinogen *in vitro* confirmed that these proteins could bind directly, and that this interaction was both saturable and inhibitable. Lysin bound both the Aα and Bβ chains of fibrinogen, but not the γ subunit. Binding of lysin to the Bβ chain was further localized to a region within the fibrinogen D fragment. Disruption of the SF100 *lysin* gene resulted in an 83±3.1% reduction (mean ± SD) in binding to immobilized fibrinogen by this mutant strain (PS1006). Preincubation of this isogenic mutant with purified lysin restored fibrinogen binding to wild type levels. When tested in a co-infection model of endocarditis, loss of lysin expression resulted in a significant reduction in virulence, as measured by achievable bacterial densities (CFU/g) within vegetations, kidneys, and spleens. These results indicate that bacteriophage-encoded lysin is a multifunctional protein, representing a new class of fibrinogen-binding proteins. Lysin appears to be cell wall-associated through its interaction with choline. Once on the bacterial surface, lysin can bind fibrinogen directly, which appears to be an important interaction for the pathogenesis of endocarditis.

## Author Summary

The binding of bacteria to human platelets is thought to be a central event in the development of endocarditis (a life-threatening cardiovascular infection). We have previously found that platelet binding by *Streptococcus mitis* is mediated by surface components encoded by a bacteriophage contained within the host bacterium. We now show that lysin (an enzyme of bacteriophage origin) contributes to platelet binding via its direct interaction with fibrinogen on the platelet surface. Lysin bound to purified fibrinogen *in vitro*, and this interaction specifically involved the Aα and Bβ chains of fibrinogen. Binding of lysin to the Bβ chain was further localized to a region within the fibrinogen D fragment. Disruption of the gene encoding lysin gene resulted in a significant reduction in binding to fibrinogen by *S. mitis*, as well as a major reduction in virulence, as measured by a rat model of endocarditis. These results indicate that lysin is a multifunctional protein, representing a new class of fibrinogen-binding molecules. Lysin is localized to the bacterial surface via its interaction with cell wall choline, where it then can bind fibrinogen directly. Cell surface lysin apparently also contributes to the development of endovascular infections via its previously unrecognized fibrinogen binding activity.

## Introduction

The pathogenesis of infective endocarditis is a complex process, involving numerous host-pathogen interactions [1], [2]. A key interaction for disease establishment and progression is the binding of microbes to human components, including platelets, fibrinogen, fibrin, and fibronectin [3], [4], [5], [6], [7], [8]. Although this binding appears to be a central requirement for virulence, only a limited number of endocarditis-related adhesins has been identified [7], [8], [9].

Among the viridans group streptococci, *Streptococcus mitis* is a leading cause of endovascular infection [10], [11], [12], [13], [14]. Despite its increasing importance as a human pathogen, relatively little is known about the virulence determinants of this organism, particularly with regard to its interaction with platelets or other host components. Our previous studies identified two surface proteins (PblA and PblB) encoded by a lysogenic bacteriophage (SM1) that mediate the binding of *S. mitis* to human platelets, through their interaction with the membrane ganglioside GD3 [15], [16], [17]. Disruption of the genes encoding PblA and PblB results in a significant decrease in platelet binding *in vitro*, as well as a marked reduction in virulence, as measured by an animal model of endocarditis [16], [17].

Expression of these proteins on the bacterial surface is dependent upon the activities of phage holin and lysin, which permeabilize the cell envelope, thereby permitting the transport of PblA and PblB to the cell wall, where they attach to phosphocholine (PC) residues [16]. Of note, disruption of the gene encoding lysin (*lys)* resulted in a profound reduction in platelet binding, to levels that were significantly lower than those seen with either the parent strain, or a *pblA*/*plbB* double knock-out mutant [16]. These findings suggested that lysin mediates platelet binding in part through a mechanism independent of its role in the export of PblA and PblB.

For these reasons, we investigated the mechanisms by which lysin mediates binding to platelets, and whether this interaction contributes to the pathogenesis of streptococcal endocarditis. Our studies indicate that phage lysin can be localized on the bacterial surface through its interaction with PC residues. Surface-bound lysin can subsequently bind both free and platelet-associated fibrinogen, through its specific interaction with the Aα and Bβ chains of the protein. Loss of lysin expression is associated with reduced virulence in the setting of endocarditis, indicating that the binding of lysin to fibrinogen is an important factor in the pathogenesis of this infection.

## Results

### Characterization of lysin_SM1_ from bacteriophage SM1

**Figure 1:**
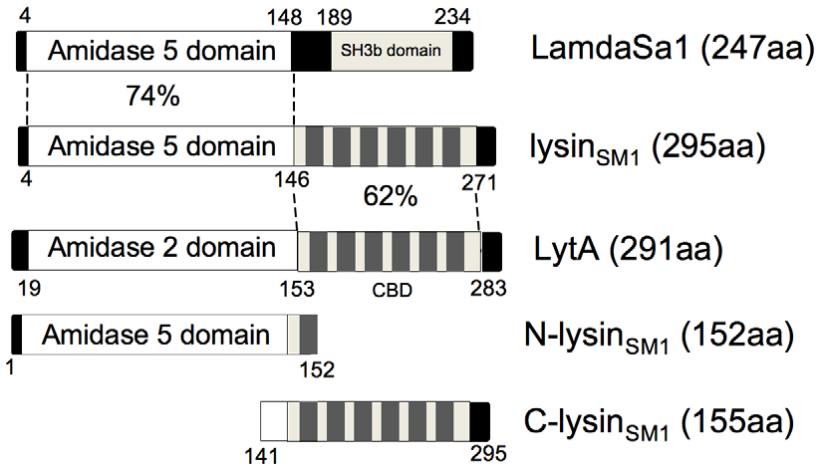
Domain organization of lysin_SM1_ and its homologs. Domains of lysin_SM1_ are compared with its closest homologs, the lambdaSa1 phage lysin of *Streptococcus agalacitae* and the LytA autolysin of *Streptococcus pneumoniae*, using the NCBI BLAST search program. Levels of identity between regions are indicated. Also shown are the alignments of the amino (N-lysin_SM1_) and carboxyl terminal (C-lysin_SM1_) truncated form of lysin_SM1_. CBD: choline-binding domain. SH3b: putative bacterial cell wall-binding domain.

Using the NCBI Conserved Domain Database (CDD) search system [18], bioinformatic analysis of the predicted amino acid sequence of lysin_SM1_ (Accession number Q9AF60), revealed that an amidase-5 domain (Pfam05382; amino acids 4–146) is present at the amino terminus, and a putative choline-binding domain is found at the carboxyl terminus (COG5263; amino acids 128–271; Fig. 1). The N-terminal domain of lysin_SM1_ (N-lysin_SM1_) exhibits 75% amino acid identity to the Pal lysin (accession number O03979) of the pneumococcal bacteriophage Dp-1, and 74% identity to the lysin (accession number Q8E0W3) of the prophage lambdaSa1 of *Streptococcus agalactiae* [19], [20]. The C terminus of lysin_SM1_ (C-lysin_SM1_) contains a choline-binding domain homologous to that found in the pneumococcal LytA autolytic enzyme (62% identity), which anchors the protein to PC residues present in LTA or teichoic acids [19].

**Figure 2:**
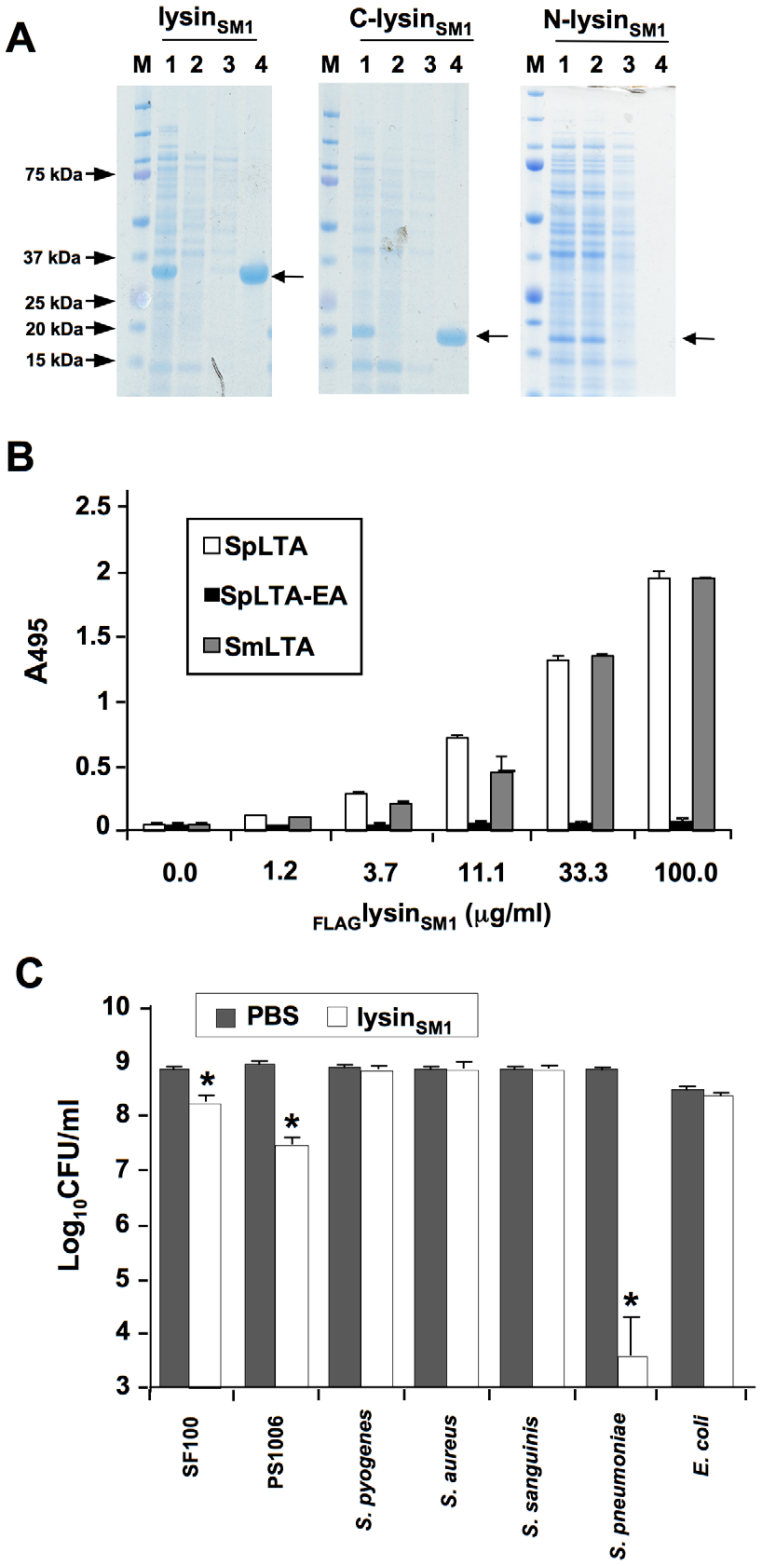
Lysin binding to phosphocholine (PC) residue and its biological activity. (A) Binding of lysin_SM1_ and its truncated forms to DEAE-cellulose. Samples were separated by SDS-PAGE and stained with Coomassie blue. M: MW standards; lane 1: whole cell extracts of *E. coli* expressing indicated protein*;* lane 2: proteins not retained by DEAE cellulose column; lane 3: proteins eluted with 1.5 M NaCl - 0.1% choline chloride; lane 4: proteins eluted with 2% choline chloride. Arrows indicate position of expressed proteins. (B) Binding of _FLAG_lysin_SM1_ to immobilized LTA from *Streptococcus pneumoniae* (SpLTA), PC-negative pneumococci (SpLTA-EA), or *Streptococcus mitis* (SmLTA). Bars indicate the means (± S.D.) of triplicate results in a representative experiment. (C) Bactericidal activity of lysin_SM1_. Values shown are number of surviving bacteria after exposure to lysin_SM1_ or buffer (mean ± S.D.). *  = *P*<0.05, compared with the same strain exposed to buffer alone.

To assess whether lysin_SMl_ demonstrated its predicted biological activities, we first examined its binding to DEAE-cellulose, a property that is a hallmark of choline-binding proteins [21]. Lysin_SM1_, N-lysin_SM1_, and C-lysin_SM1_ were expressed individually in *Escherichia coli*, and lysates from these strains were applied to a DEAE-cellulose column [16]. N-lysin_SM1_ failed to bind the matrix, with the protein being detected in the wash volumes (Fig. 2A). In contrast, lysin_SM1_ and C-lysin_SM1_ were only eluted with ™ buffer containing 2% choline chloride. Thus, lysin_SM1_ appears to be a choline-binding protein, with this interaction being mediated by the C terminus.

We then examined whether purified lysin_SM1_ bound directly to PC residues of LTA purified from *S. pneumoniae* HS0001 and *S. mitis* SF100. Purified _FLAG_lysin_SM1_ was incubated with immobilized LTAs, and binding was assessed by ELISA with anti-FLAG antibody. As shown in Fig. 2B, lysin_SM1_ bound LTA from *S. mitis* SF100 and from *S. pneumoniae* HS0001, both of which contain PC. Of note, binding levels of lysin_SM1_ to these LTAs were comparable and concentration-dependent. In contrast, little or no binding to LTA was detected from strain HS0001-EA, which has no PC. C-lysin_SM1_ also bound LTA from SF100 and HS0001, whereas N-lysin_SM1_ did not (Figure S1). These results confirm that lysin_SM1_ interacts with the PC residues of LTA, and that binding is mediated by the predicted choline-binding domain within the C terminus.

As mentioned above, analysis of the predicted amino terminus of lysin_SM1_ indicated that it encodes an amidase with g-D-glutaminyl-L-lysin endopeptidase activity [20]. To assess its lytic activity, we tested the bactericidal properties of lysin_SM1_ *in vitro* (Fig. 2C). When compared with organisms treated with buffer alone, exposure of *S. pneumoniae* HS0001 to lysin_SM1_ resulted in a mean (± S.D.) reduction of 5.07±1.28 log_10_ CFU per ml (P<0.05). Lysin_SM1_ was also active against the SM1 host strain SF100 and its isogenic variant, PS1006, though it only reduced mean titers by 0.8±0.04 (P<0.05) and 1.65±0.21 log_10_ CFU per ml (P<0.05), respectively. No bactericidal activity was seen when tested against *Staphylococcus aureus*, *Streptococcus sanguinis, Streptococcus pyogenes,* or *E. coli*. Of note, neither purified N-lysin_SM1_ nor C-lysin_SM1_ had bactericidal activity against strains HS0001 or SF100 (data not shown). Thus, lysin_SM1_ has lytic activity against PC positive strains, such as *S. mitis* and *S. pneumoniae*, though the latter species is considerably more sensitive to the enzyme. Moreover, lysin_SM1_ requires its choline-binding domain, in addition to its predicted amidase domain, for this activity.

### Binding of recombinant lysin_SM1_ to human platelets

**Figure 3:**
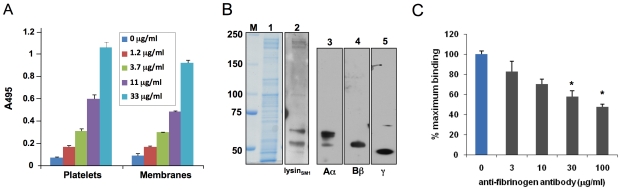
Lysin binding to platelet monolayers and platelet membranes. (A) Immobilized human platelets or platelet membranes were incubated with the indicated concentrations of purified _FLAG_lysin_SM1_, washed, and bound _FLAG_lysin_SM1_ was assessed by ELISA. Bars indicate the means (± S.D.) of triplicate data from a representative experiment. (B) Lysin_SM1_ binding to platelet membrane proteins (far Western blotting). Proteins were either stained with Coomassie blue (panel 1), or transferred to nitrocellulose membranes and probed with _FLAG_lysin_SM1_ (panel 2). Platelet membrane proteins were probed with anti-fibrinogen Aα chain (panel 3), anti-fibrinogen Bβ chain (panel 4), or anti-fibrinogen γ chain (panel 5) polyclonal antibodies. (C) Inhibition of _FLAG_lysin_SM1_ binding to platelet membranes by fibrinogen IgG polyclonal antibody. Immobilized platelet membranes were incubated with the indicated concentrations of rabbit anti-fibrinogen IgG prior to testing for binding by _FLAG_lysin_SM1_ (5 µg/ml). Bars indicate the means (± S.D.) of triplicate results from a representative experiment. *  = *P*<0.05, compared with no antibody treatment.

We have previously observed that disruption of the gene encoding lysin_SM1_ resulted in a significant reduction in platelet binding by *S. mitis* [16]. To assess whether lysin_SM1_ could interact directly with human platelets, we evaluated the binding of _FLAG_lysin_SM1_ to immobilized human platelets and to isolated platelet membranes (Fig. 3A). _FLAG_lysin_SM1_ was incubated with platelet monolayers or platelet membranes, and bound _FLAG_lysin_SM1_ was then detected with anti-FLAG antibody. When tested by this approach, we found that _FLAG_lysin_SM1_ strongly interacted with both whole platelets and platelet membranes in a concentration-dependent manner. In contrast, no binding of _FLAG_lysin_SM1_was seen to wells coated with only a casein-based blocking reagent (Western Blocking Reagent; Roche).

To identify the membrane receptor for lysin_SM1_, we assessed by far Western blotting the binding of _FLAG_lysin_SM1_ to platelet membranes that had undergone SDS-PAGE and transfer to nitrocellulose (Fig. 3B). Although the platelet membrane extracts contained numerous proteins, ranging in mass from 50 to 250 kDa, _FLAG_lysin_SM1_ bound only a small number of proteins. The highest levels of binding were seen with two proteins of MW 65 kDa and 55 kDa, which were similar to the molecular masses of the Aα and Bβ chains of human fibrinogen (64 and 56 kDa), respectively. To confirm that platelet membrane extracts contained fibrinogen, the preparations were probed with antibodies directed against the three major chains of fibrinogen (Aα, Bβ and γ). As shown Fig. 3B, each subunit of fibrinogen was present in the membrane extracts. To directly confirm that lysin_SM1_ bound fibrinogen on platelet membranes, we assessed whether lysin_SM1_ binding to immobilized platelet membranes was inhibited by anti-human fibrinogen IgG. As shown in Fig. 3C, pre-treatment of membranes with 30 or 100 µg/ml of anti-fibrinogen antibodies significantly reduced subsequent lysin binding. These results further indicate that fibrinogen is the principal component on platelet membranes and that lysin_SM1_ is bound to platelet membranes through it interaction with fibrinogen.

### Binding of lysin_SM1_ to fibrinogen

**Figure 4:**
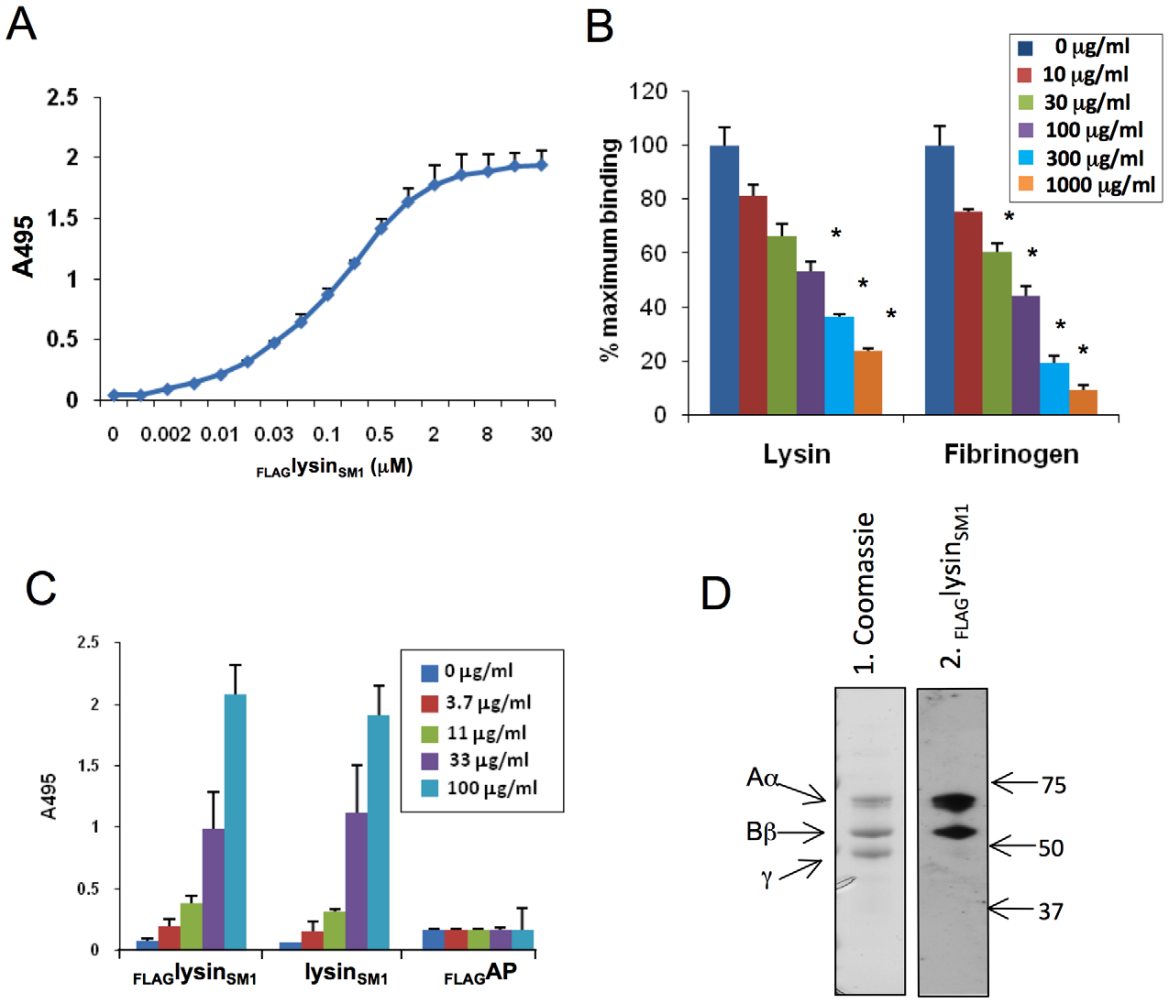
Lysin-fibrinogen binding. (A) Binding of _FLAG_lysin_SM1_ to immobilized human fibrinogen. X-axis indicates concentration of _FLAG_lysin_SM1_ incubated with fibrinogen. (B) Inhibition of _FLAG_lysin_SM1_ binding to immobilized fibrinogen by unlabelled lysin_SM1_ or fibrinogen. The binding of _FLAG_lysin_SM1_ (3 µg/ml) to immobilized fibrinogen was tested in buffer containing 0 to 1,000 µg/ml of lysin_SM1_ or fibrinogen. *  = *P*<0.05, as compared with 0 µg/ml. (C) Fibrinogen binding to immobilized _FLAG_lysin_SM1_, unlabelled lysin_SM1_, or _FLAG_AP. Concentrations indicate amount of protein added. Bars are the means (± S.D.) of triplicate results from a representative experiment. (D) Binding of _FLAG_lysin_SM1_ to fibrinogen by far Western blotting. Fibrinogen was separated by SDS-PAGE and stained with Coomassie blue (panel 1) or transferred to nitrocellulose and probed with _FLAG_lysin_SM1_ (panel 2). The three bands detected in panel 1 correspond to Aα, Bβ, γ chains of fibrinogen. Numbers indicate molecular mass. Binding of _FLAG_lysin_SM1_ to the Aα and Bβ chains was readily observed, but not to the γ chain (panel 2).

Since fibrinogen is a key factor in the pathogenesis of infective endocarditis, and because it is a receptor for some bacterial adhesins [7], [22], [23], [24], [25], [26], we further investigated the interaction of this protein with lysin_SM1_. We first assessed the binding of the increasing concentrations of _FLAG_lysin_SM1_ to human fibrinogen (3 µg/ml) immobilized in microtiter wells. In control studies, no significant binding of fibrinogen by FLAG-tagged alkaline phosphatase (_FLAG_AP) was detected (Figure S2). In contrast, _FLAG_lysin_SM1_ showed significant binding to immobilized fibrinogen, which increased in direct proportion to the amount of protein applied (Fig. 4A). At concentrations above 125 µg/ml _FLAG_lysin_SM1_, binding reached a plateau, indicating that it was saturated. In addition, the binding of _FLAG_lysin_SM1_ to immobilized fibrinogen was effectively blocked by both unlabeled lysin_SM1_ and fibrinogen (Fig. 4B). Similar levels of lysin binding were seen with rat fibrinogen, the host used for our subsequent virulence assays (Figure S3).

For some bacteria, binding to fibrinogen is dependent on whether the protein is in solution or immobilized on a surface. For example, Group A and G streptococci can bind both soluble and immobilized forms of fibrinogen, whereas several oral streptococci appear to bind only immobilized fibrinogen [27], [28], [29]. To assess whether fibrinogen binding by lysin_SM1_ was phase-dependent, we reversed the binding conditions, such that _FLAG_lysin_SM1_, untagged lysin_SM1_, and _FLAG_AP (all at 10 µg/ml) were immobilized in microtiter wells, and probed with the increasing concentration of fibrinogen in solution. Under these conditions, fibrinogen was still found to bind lysin_SM1_ and _FLAG_lysin_SM1_ comparably, whereas no significant binding to _FLAG_AP was detected (Fig. 4C).

As noted above, we found that lysin_SM1_ bound two proteins associated with platelet membranes that corresponded to the Aα and Bβ chains of fibrinogen. To confirm that lysin_SM1_ binds specifically to these subunits, we assessed by far Western blotting the interaction of lysin_SM1_ with purified human fibrinogen (Fig. 4D). When separated by SDS-PAGE under reducing conditions, fibrinogen appeared as three bands, having the expected masses. When transferred to nitrocellulose and probed with _FLAG_lysin_SM1_, binding could be detected to the Aα and Bβ chains only, confirming the results seen with platelet membranes.

### Lysin_SM1_ binds fibrinogen fragment D but not fragment E

**Figure 5:**
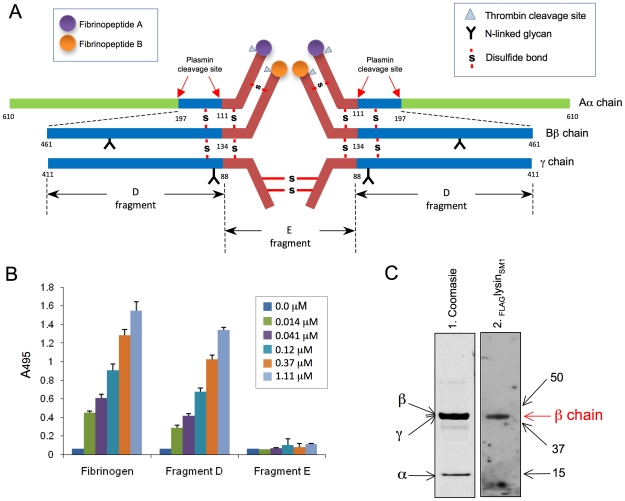
Binding of _FLAG_lysin_SM1_ to immobilized fibrinogen fragments D and E. (A) Schematic diagram of human fibrinogen. The Aα, Bβ, and γ chains, major disulfide linkages, and plasmin cleavage sites are shown. (B) Indicated concentrations of _FLAG_lysin_SM1_ were incubated with immobilized human fibrinogen, fibrinogen fragment D, or fragment E. Bound _FLAG_lysin_SM1_ was detected with anti-FLAG antibody. Bars indicate the means (± S.D.). (C) Fibrinogen fragment D was separated by SDS-PAGE under reducing conditions and stained with Coomassie blue (panel 1) or transferred onto nitrocellulose membrane and incubated with _FLAG_lysin_SM1_ (5 µg/ml). The bound proteins were detected with anti-FLAG antibody (Lane 2). Arrows indicate positions of Aα, Bβ, and γ chain fragments. Numbers indicate molecular mass (kDa).

The fibrinogen molecule is comprised of two subunits, each containing three polypeptide chains (Aα, Bβ, and γ; Fig. 5A). Cleavage of fibrinogen with plasmin produces a series of fragments, most notably the E fragment containing the central part of the molecule, and the D fragment containing the terminal regions. To further identify the domains of fibrinogen bound by lysin_SM1_, we examined the interaction of _FLAG_lysin_SM1_ to the D and E fragments. When assessed by ELISA, _FLAG_lysin_SM1_ showed high levels of binding to immobilized fragment D, which were comparable to those seen with whole fibrinogen (Fig. 5B). In contrast, no significant binding to the E fragment was seen.

Purified fibrinogen fragment D contains three subunits, each representing a part of the three major chains (α chain fragment  = 15 kDa, β chain fragment  = 44.5 kDa, and γ chain fragment  = 42 kDa) (Fig. 5A). To further identify the subdomains of fibrinogen bound by lysin_SM1_, purified fragment D was separated under reducing conditions and transferred to a nitrocellulose membrane. When assessed by far Western blotting, binding by _FLAG_lysin_SM1_, was limited to the Bβ chain component of fragment D with no binding detected to the Aα chain (Fig. 5C). These data indicate lysin_SM1_ binds a region contained within AA 134–461 of the Bβ chain. Of note, lysin_SM1_ bound the full-length Aα chain (Fig. 3B), but not its D or E fragments (Fig. 5C), suggesting that the lysin_SM1_ binding to the Aαchain requires the C terminus (AA 197–610).

### Lysin_SM1_ promotes the interaction of *S. mitis* SF100 with fibrinogen

**Figure 6:**
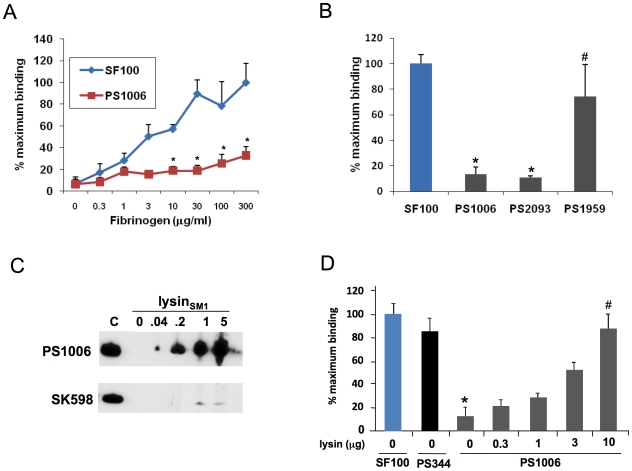
Lysin mediates binding of bacteria to fibrinogen. (A) 10^6^ CFU of SF100 or PS1006 were incubated with wells pretreated with the indicated concentrations of fibrinogen. Values represent percent of SF100 binding to wells treated with 300 µg/ml fibrinogen. (B) Complementation of the *lys* mutant (PS1006) with the *lys* gene *in trans* assessed by measuring *S. mitis* binding to immobilized human fibrinogen. PS1959 (PS1006 complemented with *lys*) demonstrated significantly greater levels of binding (P<0.05) than PS1006 or PS2093 (PS1006 complemented with pDE123 control vector). Levels of fibrinogen binding by PS1959 were comparable to those seen with SF100 (P>0.05). (C) Binding of _FLAG_lysin_SM1_ to PS1006, but not SK598. Bacteria were incubated with purified lysin_SM1_, and lysin_SM1_ bound to the cell wall was detected with anti-FLAG antibody. Lane C contains purified lysin_SM1_ (0.2 µg/ml) as a positive control. (D) PS1006 was incubated with immobilized fibrinogen in the presence of indicated concentration of purified _FLAG_lysin_SM1_ (µg/ml). Values represent percent of SF100 binding, and are the means of triplicate results from a representative experiment. *  = P<0.05 compared with SF100; #  = P>0.05 compared with PS1006.

To assess the impact of lysin expression on bacterial binding to fibrinogen, we compared the adherence of SF100 (WT) and PS1006 (Δ*lysin*) to fibrinogen immobilized in microtiter wells. As shown in Fig. 6A, SF100 had high levels of binding to immobilized fibrinogen, which increased in proportion to the amount of fibrinogen in the wells. PS1006 showed markedly reduced levels of binding, as compared with the parent strain. For example, when tested with wells coated with 30 µg/ml of fibrinogen, PS1006 had only 18.8±4.7% (mean ± SD) of maximal binding, as compared with 89.7±12.8% for SF100 (P<0.05, unpaired t-test). Complementation of the *lysin* mutation *in trans* restored fibrinogen binding by PS1006 (Fig. 6B), thereby demonstrating that the loss of binding observed with *lysin* disruption was not due to polar or pleiotropic effects.

The above results suggested that the binding to immobilized fibrinogen by SF100 is mediated by lysin_SM1_ expressed on the bacterial surface. To confirm that lysin was sufficient to mediate fibrinogen binding, we next examined whether exogenous lysin_SM1_ could attach to the cell wall of PS1006 and restore binding. The PC-negative strain SK598 served as a negative control. Each strain was incubated with purified _FLAG_lysin_SM1_ at RT for 30 min. After washing to remove nonspecifically bound protein, cell wall bound _FLAG_lysin_SM1_ was extracted with 2% choline, and the amount of _FLAG_lysin_SM1_ recovered was assessed by Western blotting. As shown in Fig. 6C, exogenous _FLAG_lysin_SM1_ could readily be detected in the cell wall extracts of PS1006, whereas no binding of _FLAG_lysin_SM1_was observed with SK598.

We then assessed whether this interaction was sufficient to enhance the binding of PS1006 to fibrinogen (Fig. 6D). PS1006 was suspended in PBS containing a range of concentrations of purified lysin_SM1_ and then tested for its binding to immobilized fibrinogen, as described above. As expected, PS1006 incubated in PBS alone showed minimal levels of binding to fibrinogen. This was not due to a loss of PblA and PblB expression, since the *pblA*/*pblB* negative strain PS344 had levels of fibrinogen binding that were similar to those of the parent strain. Exposure of PS1006 to _FLAG_lysin_SM1_ increased fibrinogen binding in a concentration-dependent manner. Indeed, 10 µg per ml of _FLAG_lysin_SM1_ was sufficient to restore PS1006 binding to levels comparable to those seen with SF100.

### Role of lysin_SM1_ in the pathogenesis of infective endocarditis

To assess the impact of lysin_SM1_ on pathogenesis, we compared the relative virulence of SF100, PS344 and PS1006 in a rat co-infection model of infective endocarditis [16], [55]. We first compared SF100 with PS344 to confirm previous results obtained in a rabbit model of infection [16]. As was observed with rabbits, disruption of *pblA* and *pblB* was also associated with attenuated virulence in rats, with PS344 having significantly reduced levels of bacteria within all tissues (Table 1). Disruption of *lysin*_SM1_ also produced a significant reduction in virulence. Rats co-infected with SF100 and PS1006 had significantly lower densities of the lysin mutant strain in vegetations (mean ± SD  = 5.07±1.50 log_10_ CFU/g) as compared with the parent strain (6.91±1.35 log_10_ CFU/g; n = 8, P = 0.009). Densities of PS1006 were also significantly reduced in kidneys (P = 0.008) and spleens (P<0.001) as compared with SF100. We then examined the relative impact on virulence of abrogated PblA and PblB expression, versus loss of lysin (Table 1). In animals co-infected with PS344 and PS1006, titers of the latter mutant were significantly reduced in all tissues examined, as compared with the former. In particular, the mean densities of PS1006 within vegetations (6.59±1.45 log_10_ CFU/g) were significantly lower than those of PS344 (8.32±0.76; n = 8; P = 0.008), as were densities within kidneys (P = 0.027) and spleens (P = 0.006). We then re-analyzed these data by comparing the ratio of PS344 to PS1006 within tissues, with the CFU of each strain normalized to the number of CFU within the inoculum (competition index) (Figure S4). When assessed by this approach, the levels of the lysin_SM1_ mutant PS1006 remained significantly reduced in all tissues, as compared with PS344. Thus, lysin_SM1_ appears to be a significant virulence determinant in the setting of infective endocarditis. Moreover, its role in pathogenesis is not due solely to any effect it may have on PblA and PblB expression. Instead, it appears to have an impact upon the development of infective endocarditis independent of these other phage-encoded proteins.

**Table 1:** Impact of lysin expression on virulence in a rat model of infective endocarditis.

Strain pairs	N	Vegetations		Kidneys		Spleens	
		Mean ± S.D. (Log_10_ CFU/g)	*P*	Mean ± S.D. (Log_10_ CFU/g)	*P*	Mean ± S.D. (Log_10_ CFU/g)	*P*
**SF100** **PS344**	8	6.19±0.845.39±1.09	<0.001	3.10±0.912.28±0.94	0.018	3.20±0.562.23±0.91	0.005
**SF100** **PS1006**	12	6.91±1.355.07±1.50	0.009	3.62±1.332.40±1.35	0.008	3.58±1.172.50±1.41	<0.001
**PS344** **PS1006**	8	8.32±0.766.59±1.45	0.008	3.89±0.772.94±1.22	0.027	3.34±0.742.25±1.32	0.006

Bacterial densities within tissues 72 h after co-infection.

## Discussion

The binding of pathogenic bacteria to platelets is thought to play a key role in the pathogenesis of infective endocarditis. This interaction may be important both for the initial attachment of bacteria to the endocardial surface, and for the subsequent formation of vegetations. Numerous endocarditis-associated pathogens have been shown to bind platelets directly *in vitro*, through a variety of mechanisms [3], [4], [7], [8], [25], [30]. The ability to bind platelets *in vitro* has been linked to virulence for several of the most common endocarditis-associated species, including *Staphylococcus aureus, Streptococcus gordonii*, and *Streptococcus sanguinis* [5], [31], [32], [33]. Previous work from our laboratory has shown that platelet binding by *S. mitis* strain SF100 is mediated in part by two proteins (PblA and PblB) encoded by the lysogenic bacteriophage SM1 [16]. The functional localization of these proteins to the cell surface requires the phage lysin (lysin_SM1_), which permeabilizes the host organism, thereby permitting the transport of PblA and PblB from the cytoplasm to the bacterial surface, and their subsequent attachment to the cell wall [16]. During the course of these studies, we noted that disruption of the gene encoding lysin_SM1_ reduced platelet binding *in vitro* more profoundly than the loss of PblA and PblB localization, indicating that lysin had a role in platelet binding beyond facilitating PblA and PblB transport. It was unknown, however, whether lysin itself could directly mediate binding, or rather, the effects of lysin on bacterial permeability led to the surface expression of other proteins (either phage or bacterial) that could enhance platelet binding.

Our current results demonstrate that lysin can bind human platelets directly through its interaction with fibrinogen. Purified lysin was found to bind fibrinogen, regardless of whether the proteins were in solution or immobilized. The binding of lysin with fibrinogen also was saturable, consistent with a receptor-ligand interaction. Lysin binding was restricted to the D fragment of the Aα and Aβ chains, further indicating that this is a specific process. This interaction appears to be important for the binding of *S. mitis* to fibrinogen, since disruption of the gene encoding lysin markedly reduced fibrinogen binding by bacteria *in vitro*. The addition of exogenous purified lysin to these mutants restored binding to WT levels, confirming that lysin can directly mediate the interaction of *S. mitis* with fibrinogen.

A number of other bacterial proteins have been shown to bind fibrinogen, including the M protein and serum opacity factor of *Streptococcus pyogenes*, FbsA of *Streptococcus agalactiae*, SdrG of *Staphylococcus epidermidis*, and several proteins of *Staphylococcus aureus* (clumping factors A and B, fibronectin binding protein A) [4], [22], [25], [30], [34]. However, none of these proteins exhibit any primary sequence homology with lysin_SM1_. The staphylococcal autolysins Aaa and Aae do resemble lysin_SM1_, in that they appear to have both enzymatic and fibrinogen binding activities *in vitro* [35], [36]. A search against the SMART and Pfam databases indicates that collectively these proteins belong to the NlpC/P60 superfamily of proteins, containing their catalytic domain that are characteristic of this group of proteins. However, the predicted catalytic activity of lysin_SM1_ (amidase 5) is different from that autolysins Aaa and Aae. Lysin_SM1_ has no sequence similarity to either the Aaa or Aae protein, and unlike these other proteins, it is a choline-binding protein. Thus, lysin appears to be a multi-functional protein that can mediate *S. mitis* binding to fibrinogen, in addition to its role in the transit of the PblA and PblB proteins to the cell surface.

Lysin_SM1_ was also associated with increased virulence in a rat model of infective endocarditis. When animals were co-infected with the parent strain SF100 and the lysin_SM1_ mutant PS1006, densities of the lysin mutant were significantly reduced within vegetations, kidneys, and spleens, as compared with the parent strain. Moreover, the virulence of PS1006 was also attenuated, when compared with its *pblA* and *pblB*-deficient isogenic variant, PS344. These results indicate that, beyond its importance for PblA and PblB expression, lysin contributes to virulence through a mechanism beyond its role in the transport of these bacteriophage-encoded adhesins. It is possible that there are other, unrecognized phage-encoded virulence factors that require lysin for export or localization. However, in view of the ability of lysin to bind fibrinogen directly (both human and rat), and that fibrinogen binding has been associated with virulence for several other adhesins [37], [38], [39], [40], [41], it is likely that this interaction of lysin with fibrinogen contributes to the pathogenesis of infective endocarditis by *S. mitis*. Given that lysin-fibrinogen binding enhances bacterial adherence to platelets *in vitro*, and that bacterium-platelet binding has been linked to virulence, it is likely that lysin-mediated binding to platelets via fibrinogen is an important pathogenetic interaction. However, it is also possible that lysin mediates streptococcal binding to fibrinogen on other surfaces, such as damaged endothelium. Finally, it is conceivable that lysin contributes to virulence through other, as yet unidentified interactions.

**Figure 7:**
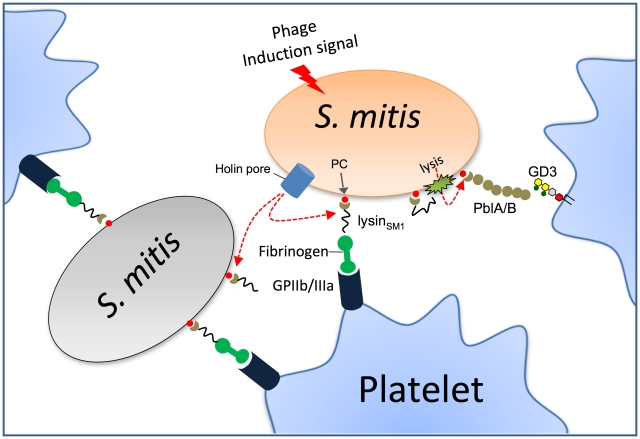
Model for the role of lysin in platelet binding. Phage lysin is exported through the holin pore and mounted on the bacterial surface of the same or adjacent organisms through its interaction with PC residues. Lysin can then mediate platelet binding via its interaction with fibrinogen and glycoprotein IIb/IIIa (GPIIb/IIIa), which is the principal fibrinogen receptor on platelets. Through its amidase activity, lysin can also permeabilize the cell wall, permitting the release and surface expression PblA and PblB. These phage proteins also interact with platelets by binding the membrane ganglioside GD3.

In summary, lysin is a novel fibrinogen-binding protein encoded by a lysogenic bacteriophage of *S. mitis*. In addition to its expected role in cell wall degradation, lysin also appears to be an adhesin mediating the attachment of this organism to human platelets, through its interaction with cell wall PC, fibrinogen, and the platelet membrane receptor for fibrinogen, glycoprotein IIb/IIIa (Fig. 7). Lysin also appears to contribute significantly to virulence, which could explain the persistence of certain bacteriophages within their host organisms. Although induction of the phage lytic cycle extracts a toll on host viability, *in vivo* this may be more than offset by the enhanced virulence resulting from lysin expression. Since fibrinogen is also present within gingival crevicular fluid, lysin-fibrinogen binding may also contribute to the colonization of oral surfaces by *S. mitis* [42], [43]. Although we do not know the exact prevalence of lysin_SM1_ homologs in other organisms, recent studies of *S. pneumoniae* and *Enterococcus faecalis* indicate that lysogenic bacteriophages encoding homologs of PblA and PblB are often present within these species [44], [45]. Since lysins are required for the phage life cycle, these findings suggest that homologs of lysin_SM1_ may also be encoded by such prophages. If so, then lysin binding to fibrinogen could prove to be an important interaction for a range of Gram-positive pathogens.

## Materials and Methods

### Ethics statement

Blood was obtained from healthy human volunteers, using a protocol approved by the Committee on Human Research at the University of California, San Francisco. All human studies were conducted according to the principles expressed in the Declaration of Helsinki. Written informed consent was obtained from all study participants prior to their participation. All procedures involving rats were approved by the Los Angeles Biomedical Research Institute animal use and care committee, following the National Institutes of Health guidelines for animal housing and care.

### Reagents

N terminal Met-FLAG-alkaline phosphatase (_FLAG_AP) and purified rat fibrinogen were purchased from Sigma-Aldrich. Purified human fibrinogen and the fibrinogen fragment D and E (produced by cleavage of fibrinogen with plasmin) were obtained from Haematologic Technologies. Rabbit anti-human fibrinogen polyclonal IgG was purchased from Innovative Research.

### Cloning and expression of lysin_SM1_

Genomic DNA was isolated from SF100, using Wizard Genomic DNA purification kits (Promega), according to the manufacturer's instructions. Polymerase chain reaction (PCR) was performed with the primers listed in Table S2. To clone *lys* gene into *E. coli* expression vector, PCR products were purified, digested, and ligated into pET28_FLAG_ to express FLAG-tagged versions of full length lysin_SM1_ (amino acids [AA] 1–295), the amino terminus of lysin_SM1_ (AA 1–158; N-lysin_SM1_), or the carboxy terminus of lysin_SM1_ (AA 141–295; C- lysin_SM1_). Untagged lysin_SM1_, C-lysin_SM1_, and His-tagged N-lysin_SM1_ (_His_N-lysin_SM1_) were cloned into pET22b(+) (Novagen). The plasmids were then introduced to *E. coli* BL21(DE3) by transformation. Lysin_SM1_, _FLAG_lysin_SM1_, C-lysin_SM1_ and _FLAG_C-lysin_SM1_ were purified with DEAE-cellulose columns, as described previously [16]. _FLAG_N-lysinSM1 and _His_N-lysin_SM1_ were purified by either Ni-NTA (Promega) or anti-FLAG M2 agarose affinity chromatography (Sigma-Aldrich), according to the manufacturers' instructions.

### Deletion or complementation of the *lysin* gene (*lys*)

A gene replacement cassette was constructed by cloning the chromosomal regions flanking *lys* upstream and downstream of the *cat* gene in pC326 [16]. A 339 bp upstream segment was amplified using primers KO4F and KO4R, and then digested with *Xho*I and *Hind*III. A 513 bp downstream segment was amplified with primers KO6F and KO6R, and then digested with *Eco*RI. The upstream and downstream fragments were cloned sequentially into the corresponding sites of pC326. The resulting plasmid, pKO-lys, was introduced into SF100 by natural transformation as previously described [17]. In brief, overnight SF100 cultures were diluted 100-fold in fresh THB supplemented with 20% heat-inactivated horse serum, 200 ng/ml competence-stimulating peptide (CSP; DWRISETIRNLIFPRRK), and 1 µg/ml of plasmid. Transformation mixtures were incubated 4 h at 37°C and then plated on blood agar containing 5 µg chloramphenicol per ml. To complement *in trans* the *lys* mutation in PS1006, *lys* was amplified using primers 3206-*Xba*I and 5206-*EcoR*I and then cloned into the streptococcal expression vector pDE123. This plasmid was derived from pDC123 by replacing the chloramphenicol resistance marker with an erythromycin resistance marker [46]. The resulting plasmid, pDE-lys, was introduced into PS1006 by natural transformation.

### Strains and growth conditions

The bacteria and plasmids used in this study are listed in Table S1. *S. mitis* strains were grown in Todd-Hewitt broth (Difco) supplemented with 0.5% yeast extract (THY). PS344 (ΔORF47-PblB::pVA891) and PS1006 (Δ*lysin_SM1_*) are isogenic variants of *S. mitis* SF100, which is an endocarditis-associated clinical isolate [16]. All three strains grow comparably well *in vitro*. *S. pneumoniae* strains were grown in either a chemically defined medium (CDM; JRH bioscience) [47] supplemented with 0.1% choline chloride, or THY. *S. pneumoniae* HS0001 is a nonencapsulated pneumococcal strain derived from the TIGR4 strain by deleting the capsule synthesis locus as described previously [48]. *S. pneumoniae* HS0001-EA is a PC-negative strain derived from HS0001 as described previously [49]. *Escherichia coli* DH5α and BL21(DE3) strains were grown at 37°C under aeration in Luria broth (LB; Difco). Appropriate concentrations of antibiotics were added to the media, if required.

### Purification of lysin_SM1_ and C-lysin_SM1_ in DEAE-cellulose

Transformed *E. coli* BL21(DE3) cells were harvested by centrifugation, washed and suspended in 50 mM Tris-maleate (™) buffer (Sigma-Aldrich), pH 6.3. Cells were disrupted by treatment with B-PER lysis solution (Pierce, Rockford, IL) and the debris was removed by centrifugation at 4,000 rpm for 10 min at 4°C. Supernatants were loaded on a 2 ml DEAE-cellulose (Sigma-Aldrich) column equilibrated with 50 mM ™ buffer, pH 6.3. The column was washed with at least 3 volumes of 50 mM ™ buffer, pH 6.3, containing 1.5 M NaCl and 0.1% choline chloride, until no protein was detected in the eluent. The retained proteins were then eluted with 50 mM ™ buffer, pH 6.3, containing 1.5 M NaCl and 2% choline chloride. Recombinant protein was dialyzed against PBS and then stored at −70°C.

### Bactericidal assay

Early log phage (A_600_ = 0.5) bacteria were harvested by centrifugation and suspended in PBS at approximately 10^8^–10^9^ CFU/ml. Bacteria samples were then incubated with or without 30 µg/ml of purified lysin_SM1_ at 37°C for 30 min. Samples were serially diluted in PBS and plated onto blood agar, to determine the number of surviving bacteria.

### Isolation of platelet membranes

Platelet membranes were prepared by glycerol lysis and gradient centrifugation, as previously described [50]. In brief, isolated human platelets were lysed in 5 volumes of lysis buffer (8.5 mM Tris-Cl, 96.5 mM NaCl, 85.7 mM glucose, 1 mM EDTA, 10 mM EGTA [pH 7.4]) containing Complete Protease Inhibitor Cocktail (Roche). The sample was centrifuged (5,900× *g*, 10 min) to remove unlysed platelets, and the supernatant was applied to a sucrose step gradient (10 ml of 33% sucrose on 5 ml of 66% sucrose in buffer). After ultracentrifugation (90 min, 63,000× *g*, 4°C), the membranes were removed, dialyzed against PBS containing 10% glycerol, and stored at −70°C.

### Far western blot analysis

Samples were separated by electrophoresis through 4–12% NuPAGE Bis-Tris gels (Invitrogen) and transferred onto nitrocellulose membranes. The membrane were treated with a casein-based blocking solution (Western Blocking Reagent; Roche) at room temperature, and then incubated for 1 h with _FLAG_lysin_SM1_ (5 µg/ml) or purified human fibrinogen (1 µg/ml) suspended in PBS-0.05% Tween 20 (PBS-T). The membranes were then washed three times for 15 min in PBS-T, and bound probe proteins were detected with mouse anti-FLAG monoclonal antibody (Sigma-Aldrich) or rabbit anti-fibrinogen polyclonal IgG antibody.

### Lysin binding to platelet monolayers or platelet membranes

Washed, fixed human platelets or purified platelet membranes were immobilized in 96 well microtiter plates as described previously [51]. To reduce non-specific adherence, the wells were then treated with the casein-based blocking reagent for 1 h at room temperature. The blocking solution was removed by aspiration, and the wells were incubated with 0 to 100 µg of _FLAG_lysin_SM1_ in PBS for 1 h, at RT, followed by washing to remove unbound protein. Bound _FLAG_lysin_SM1_ was detected by ELISA with anti-FLAG antibody. For some studies, the wells containing platelet membranes were pretreated with 0 to 100 µg/ml of rabbit anti-fibrinogen antibody for 30 min, followed by washing to remove unbound antibody. Binding by _FLAG_lysin_SM1_ (5 µg/ml) was then assessed as described above.

### Binding of recombinant _FLAG_lysin_SM1_ to fibrinogen and fibrinogen fragments

Rat fibrinogen (10 µg/ml), human fibrinogen, or human fibrinogen D or E fragments (all 15 nM in PBS), were immobilized in 96-well microtiter dishes by overnight incubation at 4°C. The wells were washed twice with PBS and blocked with 300 µl of casein-based blocking solution for 1 h at room temperature. The plates were washed three times with PBS, and a range of _FLAG_lysin_SM1_ concentrations in PBS with Tween 20 (0.05%) were added. The plates were then incubated for 2 h at 37°C. Unbound protein was removed by washing with PBS, and plates were incubated with mouse anti-FLAG antibodies for 1 h at 37°C. Binding was assessed by ELISA, using HRP-conjugated rabbit anti-mouse IgG, for 1 h at 37°C. _FLAG_AP (25–100 µg/ml) served as a control for nonspecific binding.

To examine the binding of fibrinogen to immobilized _FLAG_lysin_SM1_, untagged lysin_SM1_, or _FLAG_AP (10 µg/ml in PBS) were immobilized in 96 well microtiter plates, followed by blocking of the wells with the casein blocking solution. The wells were incubated with a range of human fibrinogen for 1 h at room temperature, followed by washing. Bound fibrinogen was detected by ELISA, using anti-human fibrinogen IgG.

### Assay for lysin_SM1_ binding to bacterial cell walls

Cultures of PS1006 and *S. mitis* SK598 in the early log phage of growth (A_600_ = 0.5) were harvested by centrifugation and suspended in PBS. The bacteria were incubated with purified _FLAG_lysin_SM1_ (0 to 10 µg/ml) for 30 min at room temperature. The samples were washed twice with PBS to remove unbound _FLAG_lysin_SM1_ and incubated with PBS-2% choline chloride to elute choline-binding proteins from the cell walls, as described previously [21]. Eluted cell wall proteins were harvested by centrifugation and loaded onto SDS-PAGE. Cell wall bound _FLAG_lysin_SM1_ was detected by western blotting with anti-FLAG antibody.

### Assay for the binding of SF100 to immobilized fibrinogen

Overnight cultures of *S. mitis* SF100 or its isogenic mutants (PS1006 and PS344) were diluted 1∶10 in fresh THY broth, incubated for 1 h at 37°C, and then exposed to UV light (λ = 312 nm) for 3 min, to induce the expression of the lysogenic bacteriophage SM1. The cultures were then incubated at 37°C for an additional 2 h, followed by harvesting by centrifugation. The pellets were suspended in PBS, and adjusted to a concentration of 10^6^ CFU/ml. One hundred microliters of each suspension were added to wells that had been coated overnight with 30 µg/well of fibrinogen in carbonate buffer. The plates were incubated at room temperature for 1 h, and the wells were washed three times with PBS to remove nonadherent bacteria. The wells were then treated with 50 µl of trypsin (2.5 mg/ml) for 30 min at 37°C to release the bound bacteria. The number of bound bacteria was determined by plating serial dilutions of the recovered bacteria onto blood agar.

### Lipoteichoic acid (LTA) purification

LTA was prepared from *S. pneumoniae* HS0001 and *S. mitis* strains by organic solvent extraction and octyl-Sepharose chromatography, as previously described [52]. In brief, bacteria were cultured at 37°C for 10 h in CDM with 0.1% choline chloride (Fisher scientific Inc.). To purify PC negative LTA, *S. pneumoniae* HS0001-EA was cultured for 16 h in CDM supplemented with 2% ethanolamine. Pelleted bacteria were suspended in 0.05 M sodium acetate (pH 4.0) and lysed by sonication. After extraction from the lysate with a chloroform and methanol mixture (1∶0.9), the LTA was adsorbed onto an octyl-Sepharose CL-4B (Sigma-Aldrich) equilibrated in a mixture of 15% n-propanol and 0.05 M sodium acetate (pH 4.7). The absorbed LTA was then eluted with 35% n-propanol in 0.05 M sodium acetate (pH 4.7).

### Analysis of LTA structure

Purified LTA was analyzed by matrix-assisted laser desorption ionization-time of flight (MALDI-TOF) mass spectrometry [52] (Figure S5). In brief, 1 µl of a sample (1 µg/ml) and 1 µl of matrix solution (0.5 M 2, 5-dihydroxybenzoic acid and 0.1% trifluoroacetic acid in methanol) were applied to a sample plate. After drying, the sample was analyzed with a mass spectrometer (Voyager Biospectrometry DE Pro workstation; PerSeptive Biosystems). Purified LTA showed three major peaks that corresponded to LTA with five, six, and seven repeating units, respectively. The mass difference between the major peaks was 1299 or 1100 amu, corresponding to an oligosaccharide repeating unit with two PC groups or two phosphoethanolamine groups [52]. In addition, PC expression by strains HS0001 and SF100 was directly assessed by western blotting with anti-PC antibody (TEPC-15; Sigma-Aldrich) [53] (Figure S6).

### Rat model of infective endocarditis

The relative virulence of SF100 and its isogenic variants was compared in a competition model of infective endocarditis, as described previously [16], [54]. In brief, Sprague-Dawley female rats (250 to 300 g each) were first anesthetized with ketamine (35 mg/kg) and xylazine (10 mg/kg). A sterile polyethylene catheter was surgically placed across the aortic valve of each animal, such that the tip was positioned in the left ventricle, to induce the formation of sterile vegetations (nonbacterial thrombotic endocarditis). The catheters were left in place throughout the study. Seven days post-catheterization, the animals were infected intravenously with an inoculum of 10^5^ CFU containing a 1∶1 mixture of a) SF100 and PS344, b) SF100 and PS1006, or c) PS344 and PS1006. At 72 hr post-infection, the rats were euthanized with thiopental (100 mg IP). Animals were included in the final analysis only if the catheters were correctly positioned across the aortic valve at the time of sacrifice, and if macroscopic vegetations were visible. All cardiac vegetations, as well as samples of the kidneys and spleens, were harvested, weighed, homogenized in saline, serially diluted, and plated onto 8% Todd Hewitt agar (±2.5 µg/ml of chloramphenicol or 5 µg/ml of erythromycin) for quantitative culture. The plates were cultured for 48 h at 37°C, and bacterial densities were expressed as the log_10_ CFU per gram of tissue. Differences in means were compared for statistical significance by the paired t-test. The data were also analyzed by calculating a “competition index,” which was defined as the ratio of the paired strains within tissues for each animal, normalized by the ratio of organisms in the inoculum. The mean of the log_10_ normalized ratios was tested against the hypothesized ‘no effect’ mean value of 0, as described previously, using a paired t-test, with P<0.05 as the threshold for statistical significance [55].

### Data analysis

Data expressed as means ± standard deviations were compared for statistical significance by the paired or unpaired *t* test, as indicated.

## Supporting Information

Figure S1 Binding of _FLAG_N-lysin_SM1_ or _FLAG_C-lysin_SM1_ to immobilized LTA. A. Binding of _FLAG_N-lysin_SM1_ or _FLAG_C-lysin_SM1_ to immobilized LTA from *Streptococcus mitis* SF100 (10 µg/ml). B. Binding of _FLAG_C-lysin_SM1_ to immobilized LTA from *Streptococcus pneumoniae* HS0001 (SpLTA-PC), which contains PC, or LTA from *Streptococcus pneumoniae* HS0001-EA (SpLTA-EA), which lacks PC. Bars indicate the means (± S.D.) of triplicate results in a representative experiment.
(0.22 MB TIF)

Figure S2 Binding of _FLAG_lysin_SM1_ or FLAG-tagged alkaline phosphatase (_FLAG_AP) to immobilized fibrinogen. Indicated concentrations of _FLAG_lysin_SM1_ or _FLAG_AP were incubated with fibrinogen immobilized in microtiter wells, as described in the Methods section. Bars indicate means ± S.D
(0.14 MB TIF).

Figure S3 Binding of _FLAG_lysin_SM1_ to immobilized rat fibrinogen. Microtiter wells were coated with rat fibrinogen (10 µg/ml), washed, and then incubated with the indicated concentrations of _FLAG_lysin_SM1_. Binding was assessed as described for human fibrinogen in the Methods section. Values shown are the means (± S.D.) of triplicate data from a representative experiment.
(0.13 MB TIF)

Figure S4 Impact of lysin_SM1_ expression on virulence. Endocarditis was produced in rats, using an inoculum containing SF100 and PS344, SF100 and PS1006, or PS344 and PS1006 at a 1∶1 ratio. 72 h post infection, the animals were sacrificed, and log_10_ CFU/g of tissue (vegetation, kidney, and spleen) for each strain was determined by plating onto selective media. *In vivo* competition index (CI) was calculated for each pair of organisms as described in the Methods. Circles represent data from individual animals. A CI below 10^0^ indicates a competitive disadvantage for A) PS344 versus PS344, B) PS1006 versus SF100, or C) PS1006 versus PS344.
(0.20 MB TIF)

Figure S5 Structural analysis of lipoteichoic acid by MALDI-TOF mass spectrometry. A. Biochemical structure of *S. mitis* and *S. pneumoniae* LTA. B. Mass spectra of *S. pneumoniae* HS0001 LTA. Peaks at *m/z* 8572, 9872, and 11171 indicate LTA molecules with 6, 7, and 8 repeating units. C. Mass spectra of *S. mitis* SF100 LTA. Peaks at *m/z* 7273, 8573, and 9872 indicate LTA molecules with 6, 7, and 8 repeating units. Due to composition of lipid tails, each peak has at least three satellite peaks that differ from the major peak by 26–28 AMU.
(0.30 MB TIF)

Figure S6 Detection of LTA phosphocholine (PC) residues. Samples (1 µg) were separated by electrophoresis through 4–12% NuPAGE Bis-Tris gels (Invitrogen) and transferred onto nitrocellulose membranes. LTA were detected with mouse anti-PC monoclonal antibody (TEPC-15; Sigma-Aldrich).
(0.34 MB TIF)

Table S1 Strains and plasmids
(0.06 MB DOC)

Table S2 Primers.
(0.05 MB DOC)
