# Supplementary figures and images for: Bacteriophage Lysin Mediates the Binding of Streptococcus mitis to Human Platelets through Interaction with Fibrinogen
Source: PLoS Pathog. 2010 Aug 12;6(8):e1001047. doi: 10.1371/journal.ppat.1001047 (PMC2920869; doi:10.1371/journal.ppat.1001047)

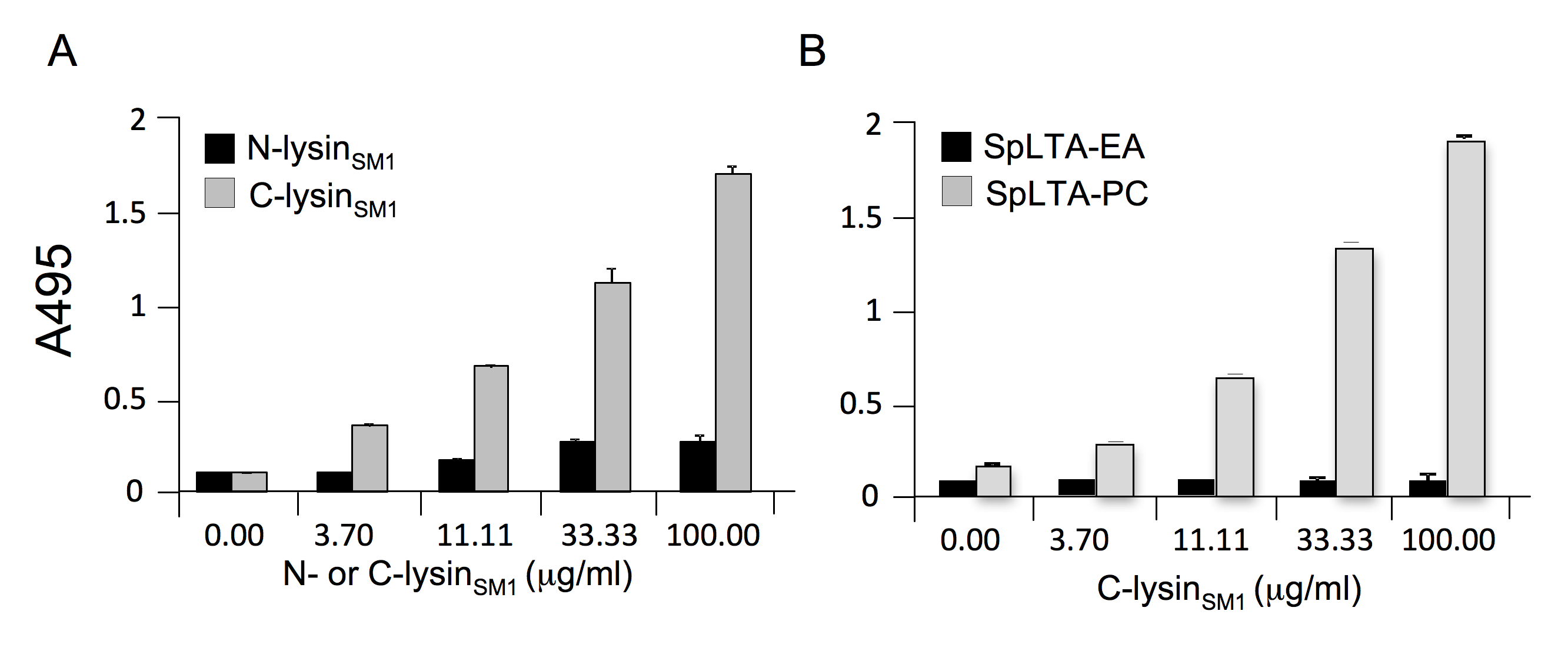

Supplement: Figure S1 — Binding of FLAGN-lysinSM1 or FLAGC-lysinSM1 to immobilized LTA. A. Binding of FLAGN-lysinSM1 or FLAGC-lysinSM1 to immobilized LTA from Streptococcus mitis SF100 (10 µg/ml). B. Binding of FLAGC-lysinSM1 to immobilized LTA from Streptococcus pneumoniae HS0001 (SpLTA-PC), which contains PC, or LTA from Streptococcus pneumoniae HS0001-EA (SpLTA-EA), which lacks PC. Bars indicate the means (± S.D.) of triplicate results in a representative experiment. (0.22 MB TIF) [file ppat.1001047.s001.tif]

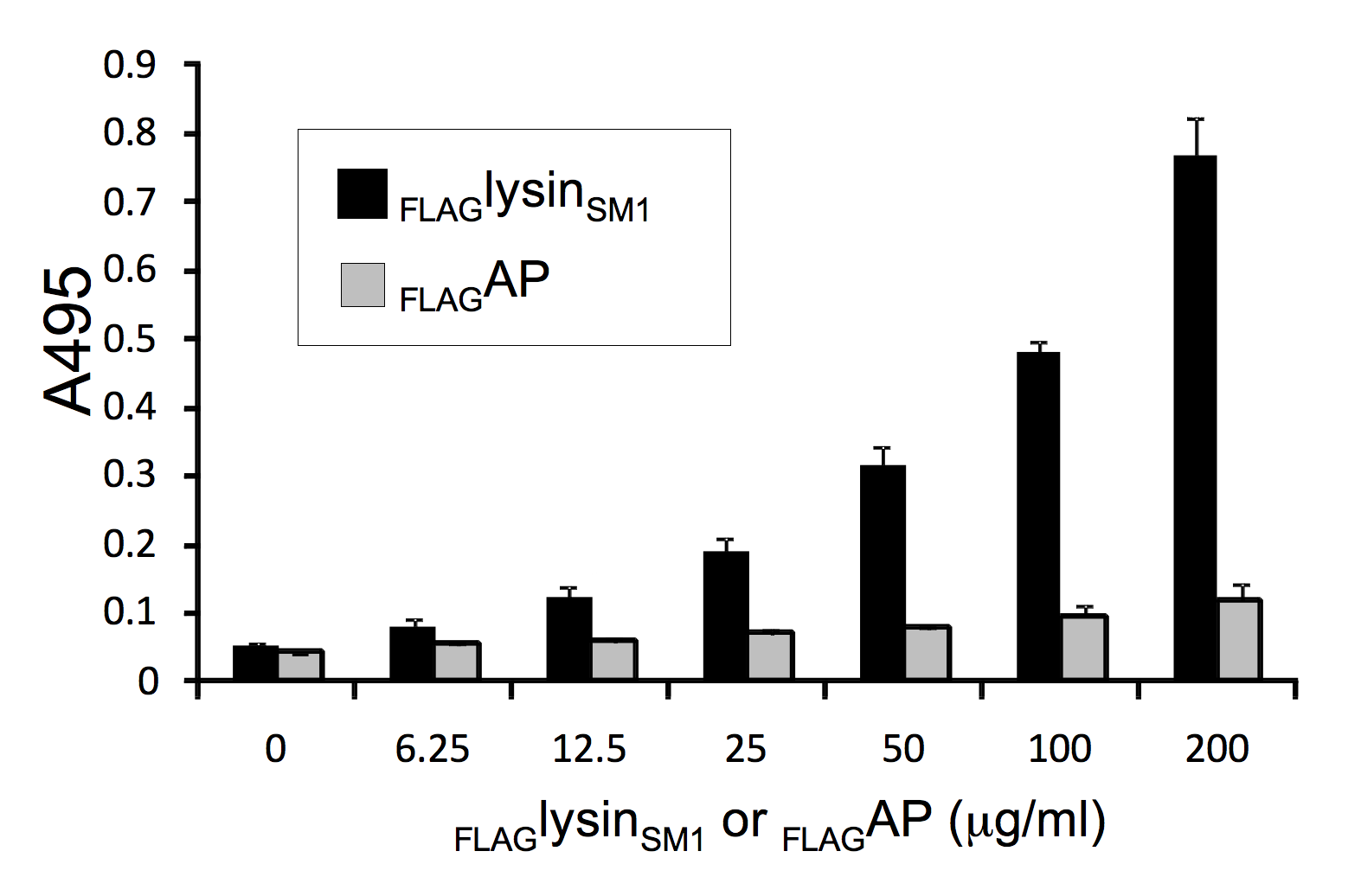

Supplement: Figure S2 — Binding of FLAGlysinSM1 or FLAG-tagged alkaline phosphatase (FLAGAP) to immobilized fibrinogen. Indicated concentrations of FLAGlysinSM1 or FLAGAP were incubated with fibrinogen immobilized in microtiter wells, as described in the Methods section. Bars indicate means ± S.D (0.14 MB TIF). [file ppat.1001047.s002.tif]

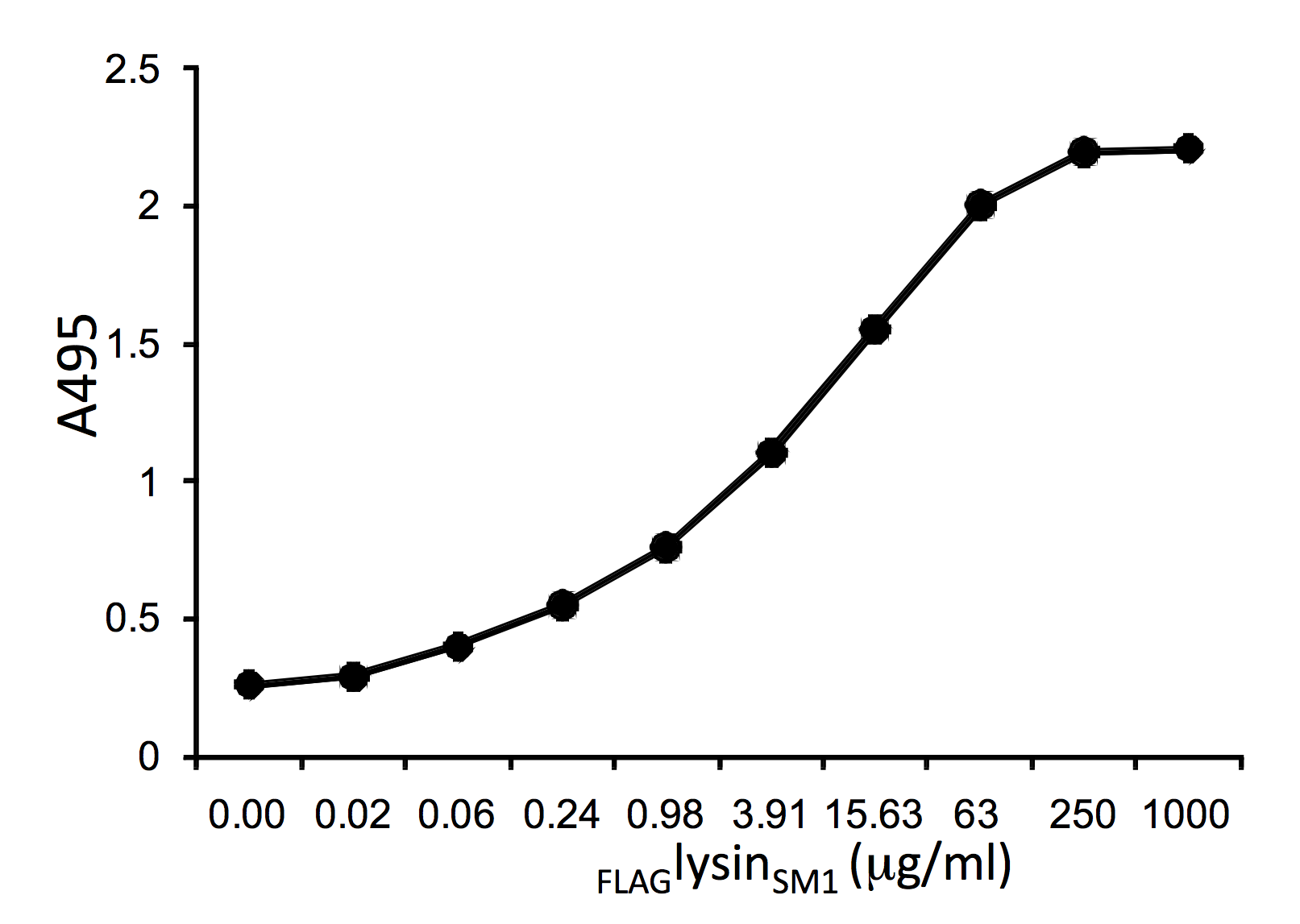

Supplement: Figure S3 — Binding of FLAGlysinSM1 to immobilized rat fibrinogen. Microtiter wells were coated with rat fibrinogen (10 µg/ml), washed, and then incubated with the indicated concentrations of FLAGlysinSM1. Binding was assessed as described for human fibrinogen in the Methods section. Values shown are the means (± S.D.) of triplicate data from a representative experiment. (0.13 MB TIF) [file ppat.1001047.s003.tif]

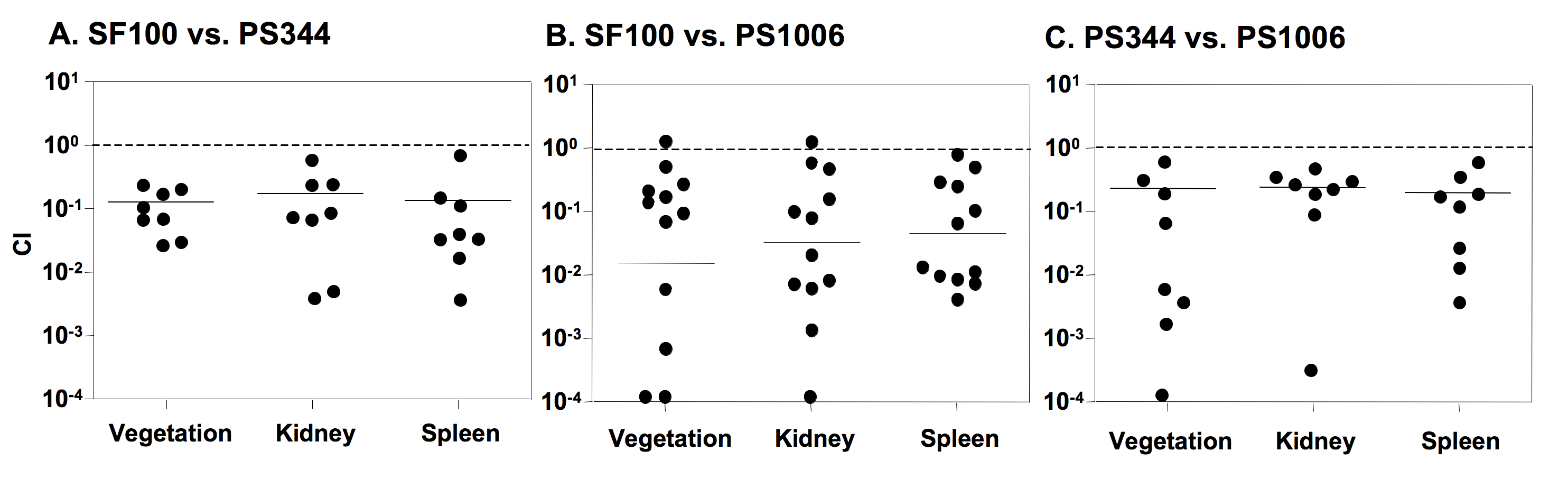

Supplement: Figure S4 — Impact of lysinSM1 expression on virulence. Endocarditis was produced in rats, using an inoculum containing SF100 and PS344, SF100 and PS1006, or PS344 and PS1006 at a 1∶1 ratio. 72 h post infection, the animals were sacrificed, and log10 CFU/g of tissue (vegetation, kidney, and spleen) for each strain was determined by plating onto selective media. In vivo competition index (CI) was calculated for each pair of organisms as described in the Methods. Circles represent data from individual animals. A CI below 100 indicates a competitive disadvantage for A) PS344 versus PS344, B) PS1006 versus SF100, or C) PS1006 versus PS344. (0.20 MB TIF) [file ppat.1001047.s004.tif]

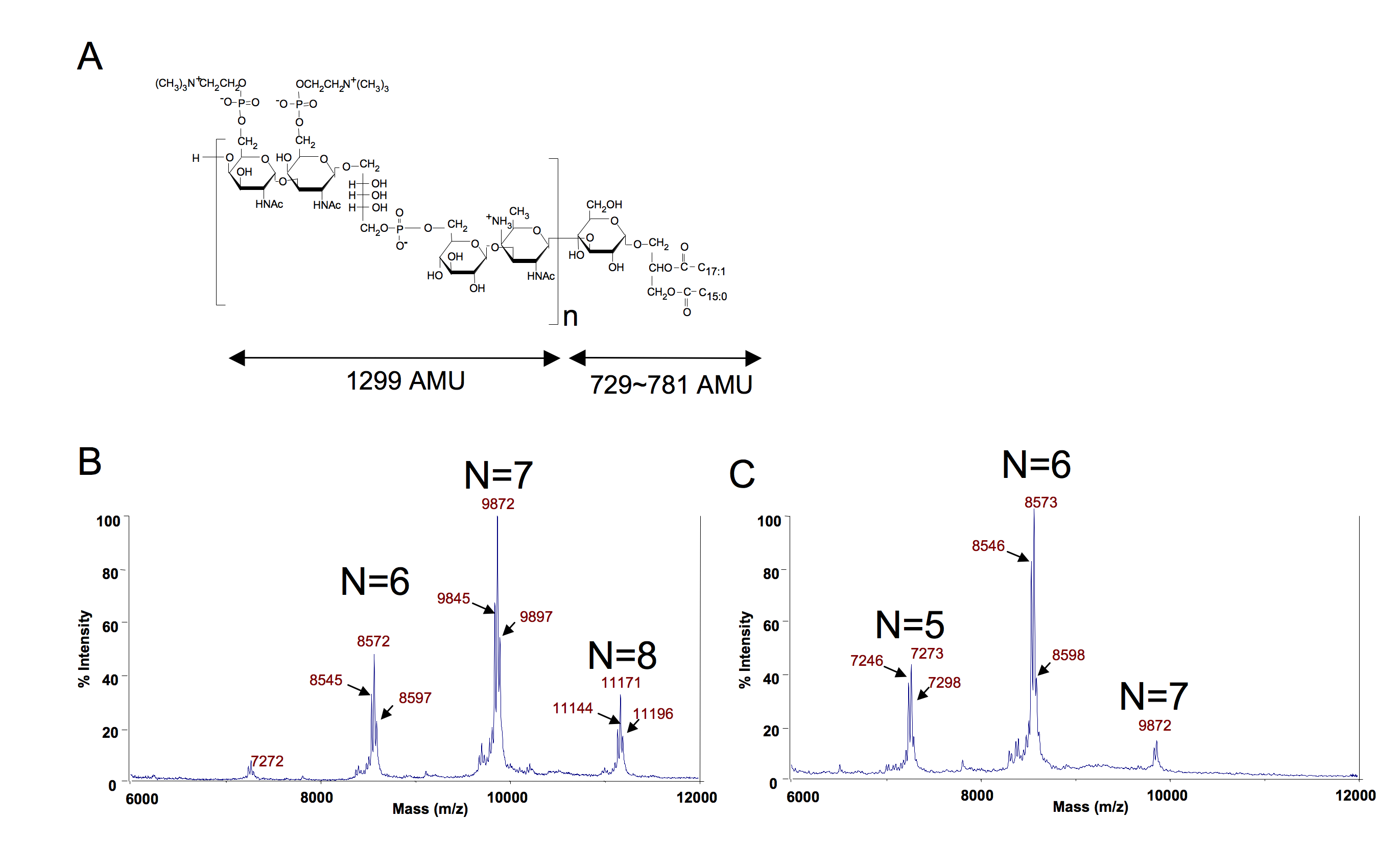

Supplement: Figure S5 — Structural analysis of lipoteichoic acid by MALDI-TOF mass spectrometry. A. Biochemical structure of S. mitis and S. pneumoniae LTA. B. Mass spectra of S. pneumoniae HS0001 LTA. Peaks at m/z 8572, 9872, and 11171 indicate LTA molecules with 6, 7, and 8 repeating units. C. Mass spectra of S. mitis SF100 LTA. Peaks at m/z 7273, 8573, and 9872 indicate LTA molecules with 6, 7, and 8 repeating units. Due to composition of lipid tails, each peak has at least three satellite peaks that differ from the major peak by 26–28 AMU. (0.30 MB TIF) [file ppat.1001047.s005.tif]

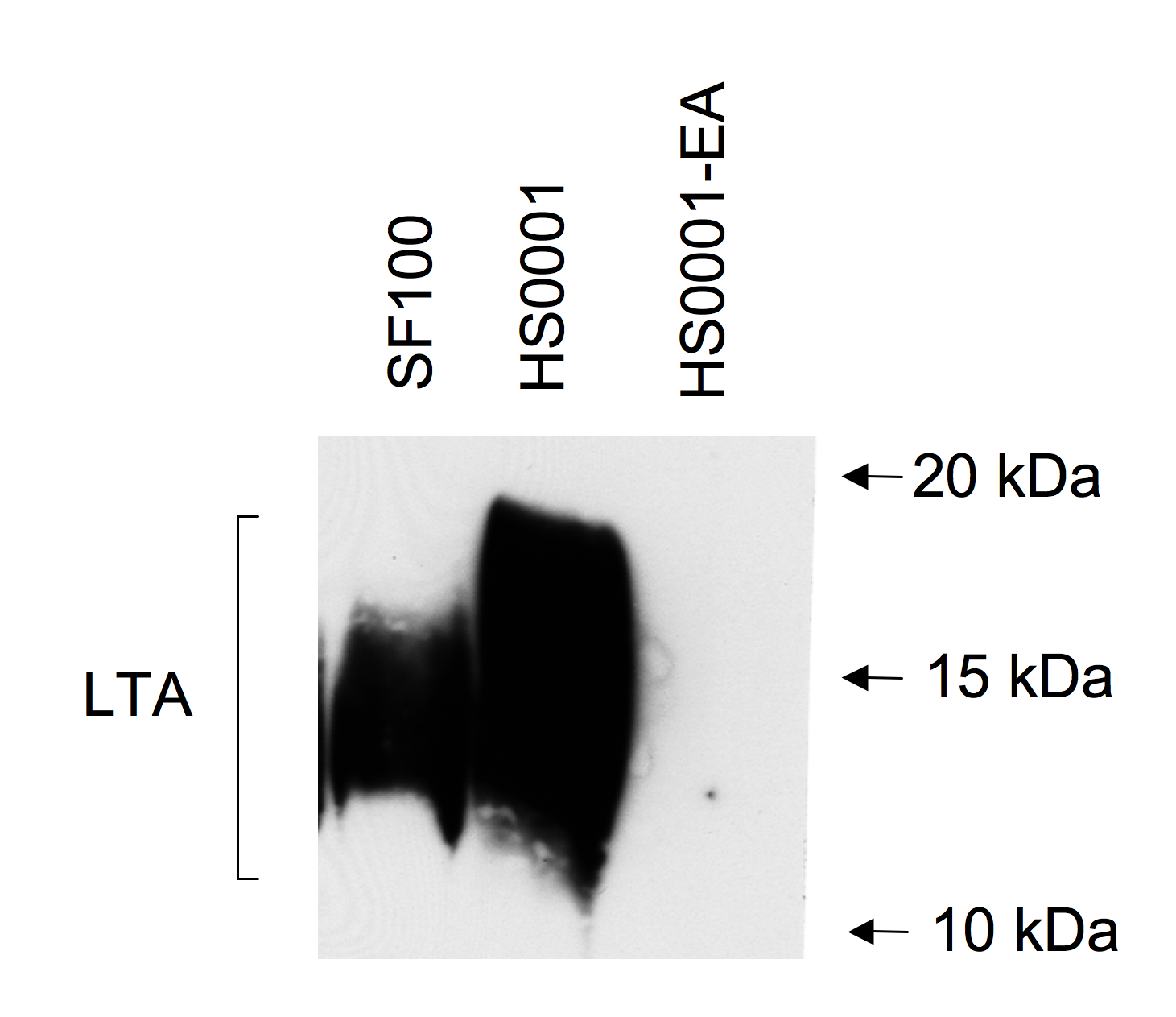

Supplement: Figure S6 — Detection of LTA phosphocholine (PC) residues. Samples (1 µg) were separated by electrophoresis through 4–12% NuPAGE Bis-Tris gels (Invitrogen) and transferred onto nitrocellulose membranes. LTA were detected with mouse anti-PC monoclonal antibody (TEPC-15; Sigma-Aldrich). (0.34 MB TIF) [file ppat.1001047.s006.tif]
