# Supplementary material for: Bacteriophage Lysin Mediates the Binding of Streptococcus mitis to Human Platelets through Interaction with Fibrinogen
Source: PLoS Pathog. 2010 Aug 12;6(8):e1001047. doi: 10.1371/journal.ppat.1001047 (PMC2920869; doi:10.1371/journal.ppat.1001047)
Supplement: Table S1 — Strains and plasmids (0.06 MB DOC) [file ppat.1001047.s007.doc]

**Table S1. Strains and plasmids**

| **Strain or plasmid** | **Genotype or description*^a^*** | | | **Source** | |
| --- | --- | --- | --- | --- | --- |
| *Escherichia coli* | | |  | | |
| DH5α | F^-^r^-^m^+^Ø80d*lacZ*∆M15 | | Gibco BRL | | |
| BL21 (DE3) | expression host, inducible T7 RNA polymerase | | Novagen | | |
| *Streptococcus mitis* | | |  | | |
| SF100 | *S. mitis;* endocarditis clinical isolate | | [1] | | |
| PS344 | SF100∆ORF47-*pblB*::pVA891, Erm^R^ | | [1] | | |
| PS1006 | SF100 ∆*lysin*::cat, Cm^R^ | | [2] | | |
| PS2093 | PS1006/pDE123 | | this study | | |
| PS1959 | PS1006/pDE123-*lys* | | this study | | |
| SK598 | PC negative strain | | [3] | | |
| *Streptococcus pneumoniae* | | |  | | |
| HS0001 | nonencapsulated strain serotype 4, PC positive | | this study | | |
| HS0001-EA | nonencapsulated strain serotype 4, PC negative | | this study | | |
| *Streptococcus sanguinis* PS478 | | | clinical isolate | | |
| *Staphylococcus aureus* RN6390 | | | [4] | | |
| Plasmids | | | | |  |
| pC326 | | Suicide vector | | | [2] |
| pDE123 | | Streptococcal shuttle vector | | | this study |
| pDE123-*lys* | | vector for expression of lysin_SM1_ | | | this study |
| pET22b+ | | expression vector | | | Novagen |
| pET28_FLAG_ | | expression vector with 3xFLAG-tag | | | this study |
| pET22-lysin_SM1_ | | vector for expression of lysin_SM1_ | | | this study |
| pET28_FLAG_lysin_SM1_ | | vector for expression of 3xFLAG-tagged lysin_SM1_ | | | this study |
| pET28_FLAG_N-lysin_SM1_ | | vector for expression of 3xFLAG-tagged N-lysin_SM1_ | | | this study |
| pET28_FLAG_C-lysin_SM1_ | | vector for expression of 3xFLAG-tagged C-lysin_SM1_ | | | this study |

^a^ Sm^r^, streptomycin resistance; Erm^R^, erythromycin resistance; Cm^R^, chloramphenicol resistance; Amp^r^, ampicillin resistance

1. Bensing BA, Rubens CE, Sullam PM (2001) Genetic loci of *Streptococcus mitis* that mediate binding to human platelets. Infect Immun 69: 1373-1380.

2. Mitchell J, Siboo IR, Takamatsu D, Chambers HF, Sullam PM (2007) Mechanism of cell surface expression of the *Streptococcus mitis* platelet binding proteins PblA and PblB. Mol Microbiol 64: 844-857.

3. Bergstrom N, Jansson PE, Kilian M, Skov Sorensen UB (2003) A unique variant of streptococcal group O-antigen (C-polysaccharide) that lacks phosphocholine. Eur J Biochem 270: 2157-2162.

4. Cheung AL, Ying P (1994) Regulation of alpha- and beta-hemolysins by the *sar* locus of *Staphylococcus aureus*. J Bacteriol 176: 580-585.
