# Supplementary material for: Bacteriophage Lysin Mediates the Binding of Streptococcus mitis to Human Platelets through Interaction with Fibrinogen
Source: PLoS Pathog. 2010 Aug 12;6(8):e1001047. doi: 10.1371/journal.ppat.1001047 (PMC2920869; doi:10.1371/journal.ppat.1001047)
Supplement: Table S2 — Primers. (0.05 MB DOC) [file ppat.1001047.s008.doc]

**Table S2. Primers**

| **Primer name** | **Sequence (5'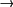3')** | **Source** |
| --- | --- | --- |
| KO4F-*Xho*I | CCG CTC GAG ATG CAA CAG ATT AAC GAA ATT TTA T | [1] |
| KO4R-*Hind*III | CTC AAG CTT TTA CTC CCC TTT CCA GGC TTC GTT | [1] |
| KO6F-*EcoR*I | GTG GAA TTC GAC ATT TGG CTC TAT GCC GGA | [1] |
| KO6R-*EcoR*I | GTG GAA TTC TTA TTT CGT TGT GAT CAA GCC GT | [1] |
| 3206-*Xba*I | GAT CTA GAT ACG TTC ATT GAG TCA G | this study |
| 5206-*EcoR*I | TTG AAT TCT TAT TTC GTT GTG ATC AAG CCG | this study |
| 3023-*Nde*I | AGT ACA TAT GGG ACT AAA TCT TG | this study |
| 3114-*Not*I | GAG CGG CCG CGG GAC TAA ATC TTG | this study |
| 3093-*Nde*I | CACATATGAATCCATCTGCAGCTGCCG | this study |
| 3145-*Not*I | GCG CGG CCG CTA ATC CAT CTG CAG C | this study |
| 5023-*Xho*I | TTT TCT CGA GTT ATT TCG TTG TG | this study |
| 5050-*Xho*I | GCC ACT CGA GTT TGA TTT CTT CGG C | this study |

1. Mitchell J, Siboo IR, Takamatsu D, Chambers HF, Sullam PM (2007) Mechanism of cell surface expression of the *Streptococcus mitis* platelet binding proteins PblA and PblB. Mol Microbiol 64: 844-857.
